# Sex differences in visuomotor tracking

**DOI:** 10.1038/s41598-020-68069-0

**Published:** 2020-07-17

**Authors:** James Mathew, Guillaume S. Masson, Frederic R. Danion

**Affiliations:** 1 Institut de Neurosciences de la Timone UMR 7289, CNRS and Aix Marseille Université, Faculté de Médecine de la Timone, 27 Bd Jean Moulin, 13005 Marseille, France; 2 Present Address: Institute of Communication Technology, Electronics and Applied Mathematics, Université Catholique de Louvain, Avenue Georges Lemaitre 4-6 bte, 1348 Louvain-la-neuve, Belgium; 3 Institute of Neuroscience, Université Catholique de Louvain, 53 Avenue E Mounier, 1200 Brussels, Belgium

**Keywords:** Cognitive neuroscience, Motor control, Oculomotor system, Sensorimotor processing, Visual system

## Abstract

There is a growing interest in sex differences in human and animal cognition. However, empirical evidences supporting behavioral and neural sex differences in humans remain sparse. Visuomotor behaviors offer a robust and naturalistic empirical framework to seek for the computational mechanisms underlying sex biases in cognition. In a large group of human participants (*N* = 127), we investigated sex differences in a visuo-oculo-manual motor task that consists of tracking with the hand a target moving unpredictably. We report a clear male advantage in hand tracking accuracy. We tested whether men and women employ different gaze strategy or hand movement kinematics. Results show no key difference in these distinct visuomotor components. However, highly consistent differences in eye-hand coordination were evidenced by a larger temporal lag between hand motion and target motion in women. This observation echoes with other studies showing a male advantage in manual reaction time to visual stimuli. We propose that the male advantage for visuomotor tracking does not reside in a more reliable gaze strategy, or in more sophisticated hand movements, but rather in a faster decisional process linking visual information about target motion with forthcoming hand, but not eye, actions.

## Introduction

Sex differences have been reported in various tasks ranging from cognitive^1^, to perceptual^2^ and motor tasks^3^. Thus, incorporating sex as a biological variable is increasingly proven relevant in behavioral and cognitive neuroscience^4–6^. However, the nature of these differences remains controversial and their origin is largely unknown. Here we focus on visuo-oculo-manual tracking, a natural behavior widely used in the field of motor control^7–10^. Tracking a moving target with the hand is a neat task to investigate the online control of visually guided movements because it relies heavily on the ability of the participant to update his/her hand motor commands on the basis of ongoing visual information. Thus, studying the ability to adjust our movements on the fly using ongoing sensory information is critical to understand how ones can readily achieve many of our everyday-life activities such as slaloming between customers when shopping at the supermarket or pursuing an opponent during a soccer match. Last but not least, visuomotor tracking is also a task tailored to investigate eye-hand coordination^8,11,12^, another valuable skill useful in many real life situations.

Although there is a clear consensus for a male advantage in throwing^13–15^, some evidence for a male advantage in visuomotor tracking have been reported^16–19^ but they remain scarce and speak for further confirmation. Our primary objective was therefore to explore the robustness of sex difference in visuomotor tracking by taking advantage of a large dataset that was previously collected in our lab^20–22^. More important, we aimed at seeking for the mechanism underlying sex differences. To do so, we carefully examined whether men and women would employ similar strategies in terms of hand movements and/or gaze control. Our first hypothesis was that men could exhibit smoother and less intermittent movements than women. Examining hand movements kinematics was found highly valuable for reporting sex difference in throwing^23^, pointing^24,25^, and lifting^26^. Here, we examined several kinematic properties of complex visuomanual trajectories such as the frequency content, the complexity, smoothness, and intermittency of hand movements to produce a detailed examination of sex differences in complex, dynamical visuomotor transformations.

Next, we explored in details the properties of gaze behavior for several reasons. First, there is some evidence of a male advantage for ocular tracking^27^ as well as for saccadic and smooth pursuit performance^28^. During manual tracking, gaze is typically concerned with monitoring the target, not the cursor^29^ presumably because keeping gaze close to the target is critical for gathering relevant information for ongoing, as well as future hand movement^30^. It is therefore important to characterize whether men and women would control ocular pursuit behavior differently. Second, there is some evidence that males exhibit a better perception of visual motion direction^31^. Third, it has been proposed that males and females employ distinct gaze behavior when solving cognitive tasks such as mental rotation^32^ or the salesman problem^33^ for instances. All these observations encouraged the comparison of male and female gaze behavior as this could account for sex differences in manual tracking.

Mapping complex target trajectories with an optimal motor responses implies a series decision-making processes in order to dynamically adapt motor outputs by determining which movement or sub-movement to make and when to make them^34,35^. We reasoned that males advantages in manual tracking that were previously reported^16–19^ could be associated with faster decisional processes dynamically linking visual information of the target with forthcoming hand actions. Such hypothesis was motivated by several studies showing that men exhibit faster reaction times to visual stimuli than women^36–41^. Interestingly all previous studies suggesting a male advantage in manual tracking^16–19^ focused on the accuracy of hand movements in the spatial domain but did not characterize their precision in the temporal domain. Here, the temporal lag between hand motion and target motion would be of particular interest as it provides key information about the speed of visuomotor processing.

To explore sex difference in human visuomotor behaviors, we analyzed two large datasets in which men and women were required to track a visual target that followed an unpredictable trajectory. Participants had to move a cursor using a joystick held by the hand. By estimating the spatial and temporal accuracies of both hand and eye movements, we can tease apart between the above-mentioned hypothesis: smoother hand movements, more accurate gaze tracking or better timing in mapping target and hand/eye trajectories. Moreover, behavioral studies investigating sex differences are often limited dataset sizes. Our strategy was to perform a detailed kinematics analysis on a large database. To do so, we built a first cohort of participants by aggregating data from three previous and distinct experiments^20–22^, in which gaze behavior was monitored. We then tested the robustness of our fist results with a second cohort of new, naïve participants extracted from an ongoing project, in which we did not monitor gaze behavior.

## Methods

### Participants

The first dataset comes from three separate cohorts of participants, issued from three separate experiments^20–22^, leading to a total of 71 participants. After the exclusion of 9 participants that participated in several studies, this number dropped to 62 (31 females, 31 males). Because the mean age of the male group was higher than the female one (28.6 vs. 25.0 years), 12 additional participants (oldest males and youngest females) were discarded so as to end up with similar age groups (25 females; 25.5 ± 2.6 years of age; 25 males; 25.5 ± 3.8 years of age); note however that similar observations were obtained with the full data set (with 62 participants). The second dataset was composed of 77 new participants (51 females; 21.4 ± 2.5 years of age; 26 males; 22.4 ± 3.5 years of age) freshly recruited for an ongoing project (i.e. not published yet). Overall a total of 127 participants were screened for this project. All participants were right-handed and had a normal or corrected-to-normal vision. Most participants were students from the campus university, and were recruited through poster and website advertisement. For all experiments, sex and handedness were determined by self-report. All participants gave written consent prior to participation. The experimental paradigm of all the studies (2016-02-03-007) was approved by the Ethics Committee of Aix-Marseille Université and complied with the Declaration of Helsinki.

### Materials and procedure

**Figure 1:**
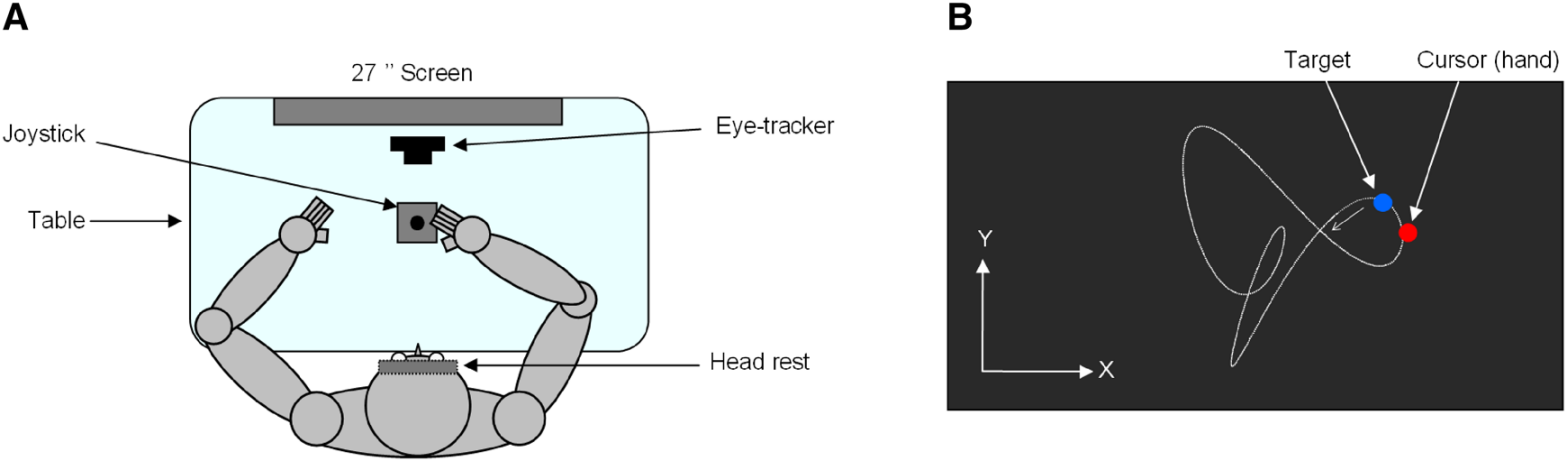
Apparatus and experimental task. (**A**) Top view of the participant sitting in the experimental setup. (**B**) Schematic view of the screen during the hand tracking task. The target trajectory (white dotted trace) and XY reference system are displayed for illustration purposes but were not visible to the participant. Adapted from Fig. 1 in Mathew et al.^22^.

Materials and procedure were similar for all cohorts. Here we decide to provide only essential information, still further details can be found in our previous papers^20–22^. Seated in a dark room, participants faced a screen (27″ inch, 144 Hz) positioned in the frontal plane 57 cm from their eyes (see Fig. 1A). Head movements were restrained by a chin and forehead rest, and a mask was positioned under the chin to prevent vision of the hands. Participants had to hold a joystick using the right hand that was positioned in line with their central sagittal plane. Eye movements were recorded via an infrared video-based eye tracker. All signals were sampled at 1,000 Hz.

### Experimental design

The tracking task (see Fig. 1B) consisted of moving the joystick with the right hand so as to keep the cursor (red disk, 0.5 cm in diameter) as close as possible to the moving target (blue disk, 0.5 cm in diameter). Participants were explicitly instructed to minimize the cursor-target distance over the whole trial, and informed that this distance would be used to assess their tracking performance. The motion of the target resulted from a combination of sinusoids: two along the frontal axis (one fundamental and a second or third harmonic), and two on the sagittal axis (same procedure). The following equations determined the target’s motion:

1 $$x_{t} = A_{1x} cos\omega t + A_{2x} {\cos}\left( {h_{x} \omega t - \varphi_{x} } \right),$$

2 $$y_{t} = A_{1y} sin\omega t + A_{2y} {\sin}\left( {h_{y} \omega t - \varphi_{y} } \right).$$

**Figure 2:**
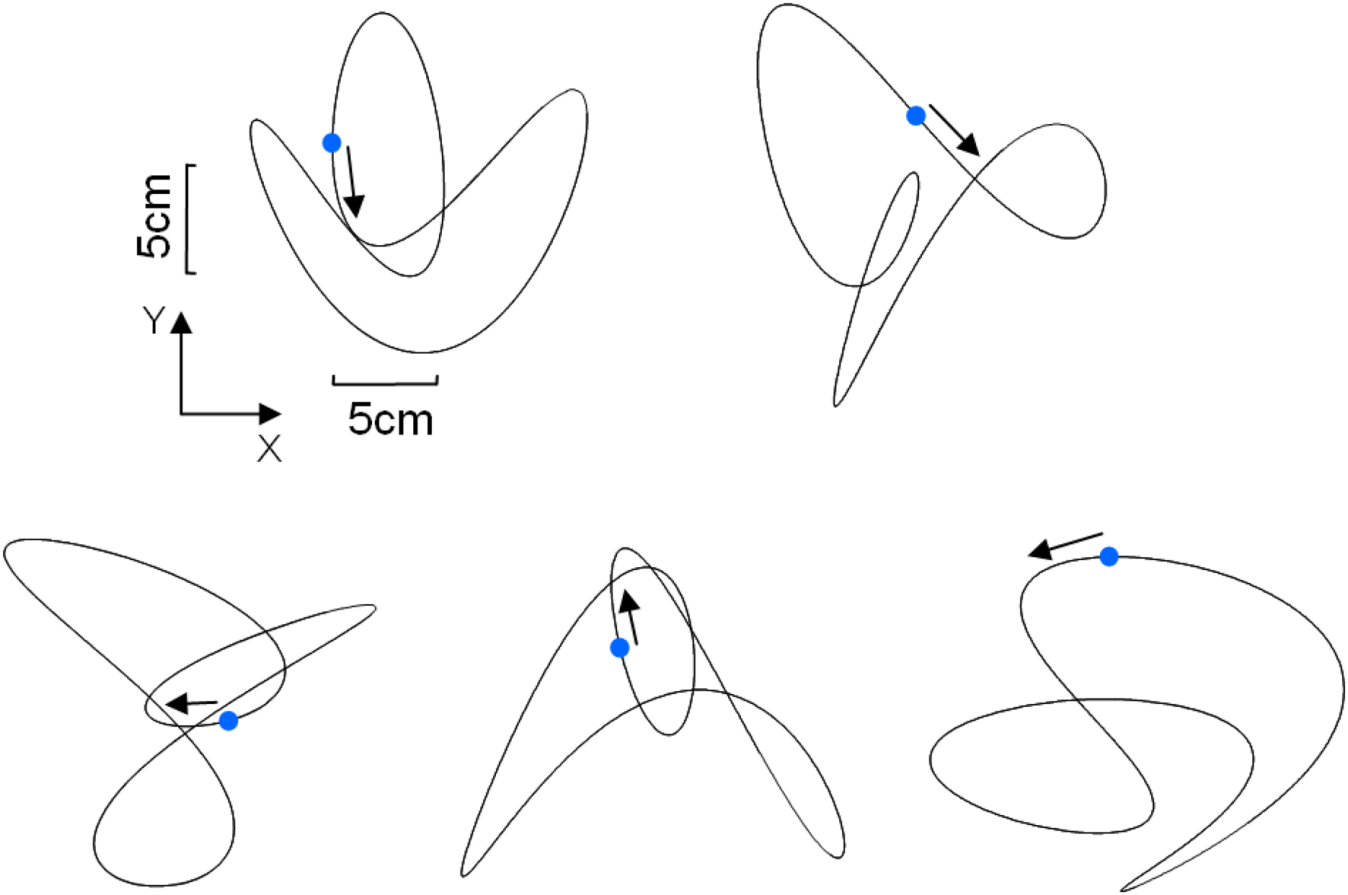
Five target paths used for manual tracking. The blue dot shows the initial position of the target, and the arrow shows its initial direction. Adapted from Fig. 3 in Mathew et al.^21^.

This technique was used to generate pseudo-random 2D patterns while preserving smooth changes in velocity and direction^29^. A total of five different patterns with a mean tangential velocity of 16 cm/s were used throughout the experiment (see Table 1 and Fig. 2). The time necessary to complete a full revolution was 5 s. Given that all trials had duration of 10 s, each movement pattern was repeated twice during each trial, and they all had a similar path length (160 cm).

**Table 1:** Target trajectory parameters. Adapted from Danion and Flanagan^29^.

Trajectory	A_1x_ (cm)	A_2x_ (cm)	${\text{h}}_{{\text{x}}}$	$\varphi_{{\text{x}}}$ (°)	A_1y_ (cm)	A_2y_ (cm)	${\text{h}}_{{\text{y}}}$	$\varphi_{{\text{y}}}$ (°)
1	5	5	2	45	5	5	3	− 135
2	4	5	2	− 60	3	5	3	− 135
3	4	5.1	3	− 60	4	5.2	2	− 135
4	5	5	3	90	3.4	5	2	45
5	5.1	5.2	2	− 90	4	5	3	22.5

As explained above the data from the first cohort were extracted from our previous experiments^20–22^. Although these experiments were carried out with separate objectives in mind, leading to various experimental conditions, all participants started by a block of ten trials to assess their baseline performance. Here we focus on the data collected during these common baseline trials. Note that during this preliminary block, the order of patterns was randomized and counterbalanced (each pattern being tested twice), and the mapping between the joystick and the cursor motion was very intuitive (right = right, left = left, forward = upward, backward = downward). Prior to the starting of experiment itself, each participant performed 2 or 3 practice trials to become familiarized with the setup and the tracking task. Regarding the second cohort, as stated previously, these participants were originally recruited for a new project in our lab, targeting different experimental conditions and the recording of hand movements only. Still, in order to further test the observation made with the first cohort, we take advantage that all these naive participants started by a block of ten baseline trials while using the same setup and procedure as previously described for the first cohort. Overall the conjunction of the two cohorts provided a total of 500 trials for eye movements, and 1,270 trials for hand movements.

### Measure and analysis

The participants’ ability to perform the tracking of the target was assessed by computing the mean distance between cursor and target position over each trial. However, such error estimation does not allow disentangling spatial and temporal errors. Therefore, the temporal relationship between cursor and target was evaluated by means of cross-correlations that simultaneously took into account the vertical and horizontal motion. To cross-correlate simultaneously horizontal (x) and vertical (y) position, x and y signals were interleaved (for more details see^29^). For each trial, the time lag corresponds to the timing shifting value at which the cross-correlation between hand and cursor movements was maximal. Correlation coefficients were never found below 0.55. Power spectral analyses of horizontal and vertical cursor motion were performed separately to provide further information about visuomotor feedback loops, known to operate within the 0–2 Hz frequency range^42,43^. Next, to provide an estimate of hand movement smoothness at each trial, we computed the mean cursor tangential velocity and its fluctuations by means of standard deviation (*SD*). To assess whether the complexity of hand motion was similar in male and female participants, approximate entropy (*ApEn*) was used as an index to characterize the unpredictability of a signal^22,44^; the larger the approximate entropy the more unpredictable the signal is. Finally, to estimate movement intermittency, we have examined how frequently hand movement alternately switched between acceleration and deceleration phases^45^. Specifically, we counted the number of zero crossings made by the cursor tangential acceleration and divided this number by 2, with the reasoning that each submovement was composed of acceleration and deceleration phase.

Regarding eye movements, the first objective consisted of evaluating how close the participant’s gaze was from the moving target. This was achieved by measuring the Euclidian distance between gaze and target, as well as the temporal lag between gaze and target using the cross-correlations technique exposed previously. A second objective was to characterize in more detail the characteristics of eye movements triggered by the participants. Using procedures described in our previous papers^22,46^, we computed for each trial the smooth pursuit gain (SP gain) and the saccadic rate. All dependent variables were averaged across the 10 trials.

### Statistics

Independent *t*-tests were used to assess the effects of SEX (male/female) for all our dependent variables. Power spectra of cursor movements were examined with two-way repeated measures ANOVAs to assess the effect of SEX and FREQUENCY (18 levels: 0.11–2 Hz with 0.11 Hz step). Lilliefors tests showed that none of the dependent variables significantly deviated from a normal distribution. The threshold of significance was always set at 0.05.

## Results

We successively present the data from the first cohort in which both eye and hand movements were recorded, and then the data from the second cohort in order to test the robustness of our findings regarding manual tracking and sex differences.

### Manual tracking is more accurate in male

**Figure 3:**
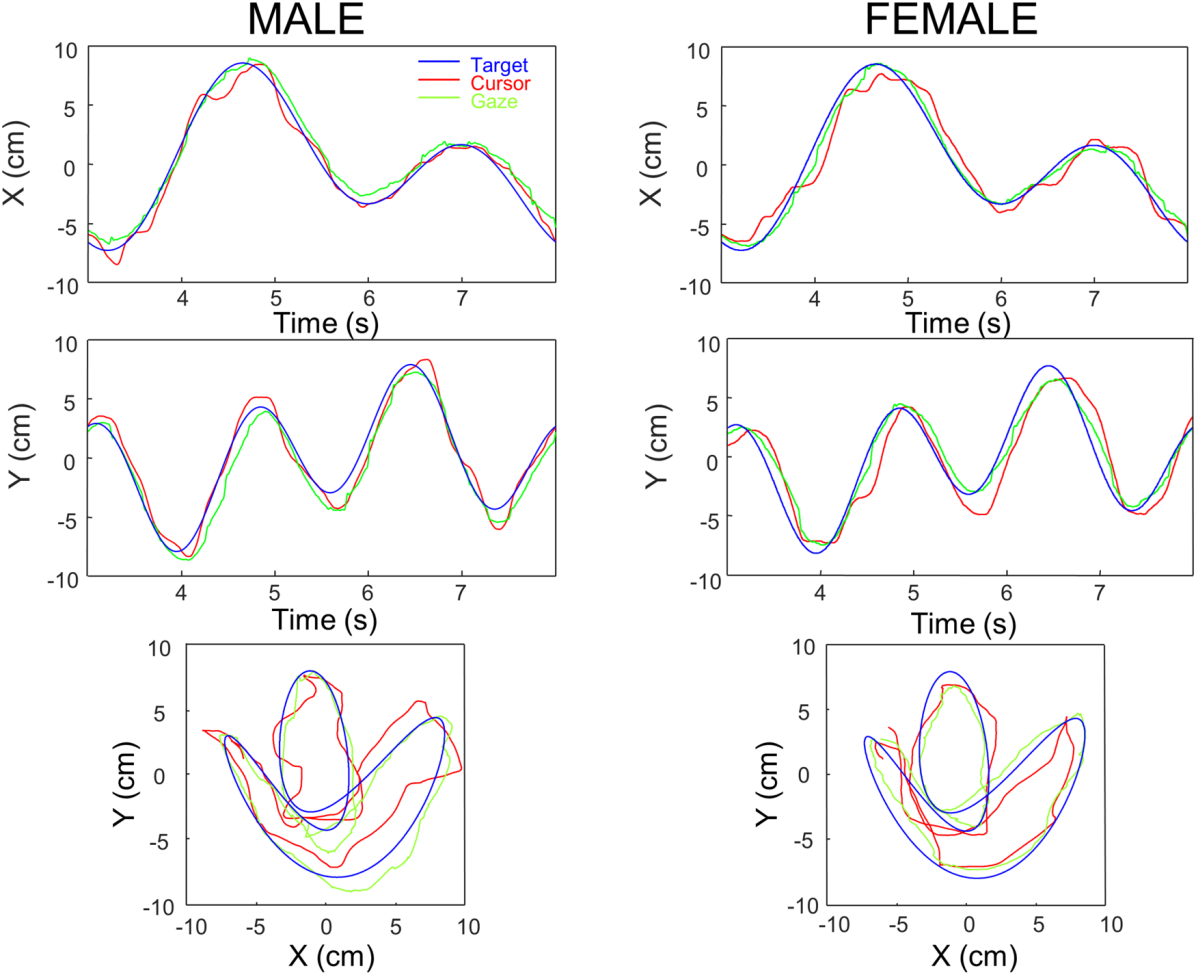
Typical portions of a trial performed by one male and one female participant. Top and middle graphs display respectively the horizontal and vertical displacement of target, cursor (hand), and gaze. Bottom graphs display the corresponding XY trajectories. For comparison purposes we have selected trials with the same target pattern.

Figure 3 plots one representative trial from one male and one female participant. As can be seen hand tracking was somewhat poorer in the female participant. In the next sections we analyze in more detail this observation over the first cohort.

**Figure 4:**
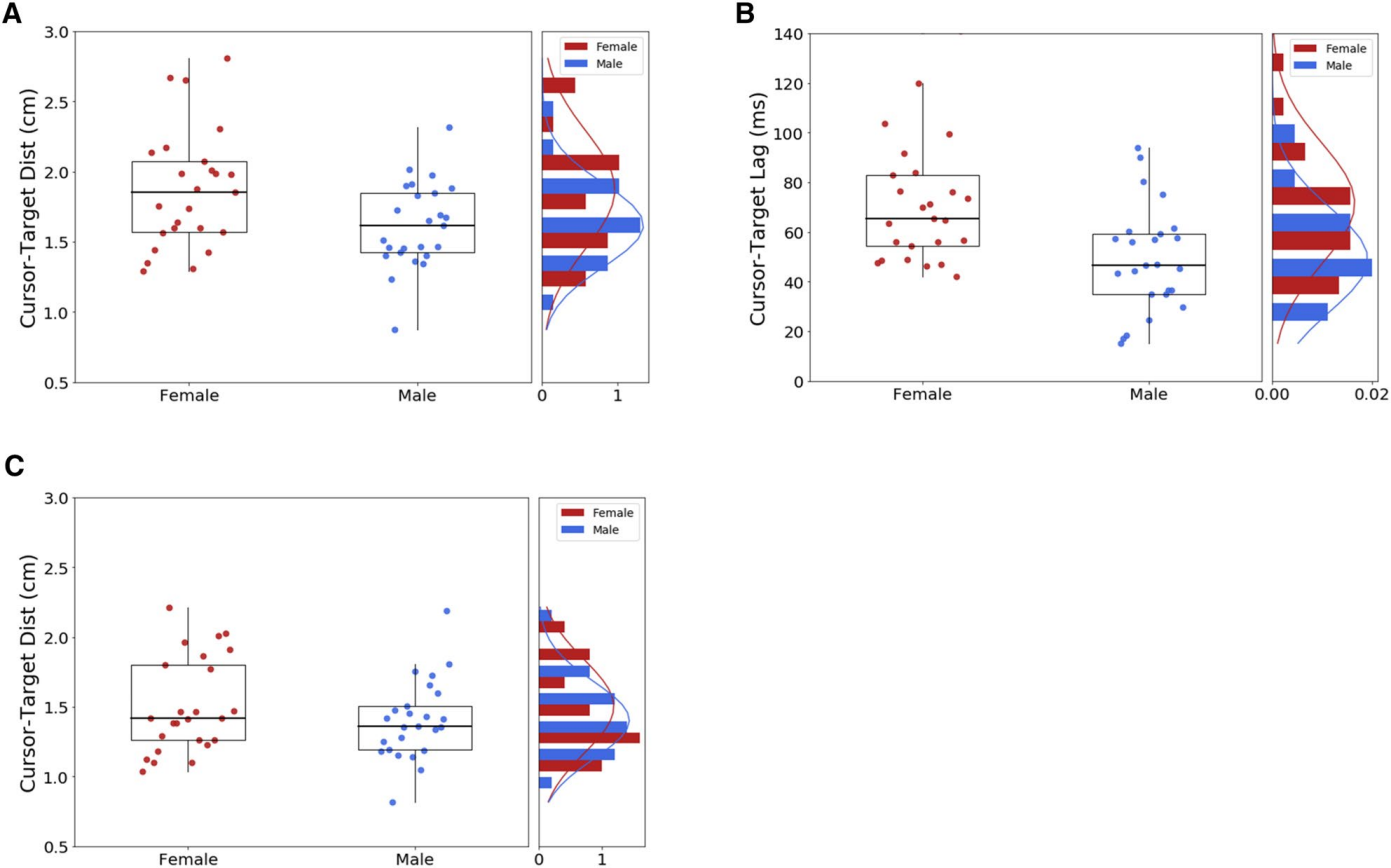
Manual tracking. (**A**) Cursor-target distance. (**B**) Cursor-target lag. (**C**) Residual cursor-target distance after compensation for cursor-target lag. Each box extends from the upper to lower quartile values of the data, with a line at the median. The whiskers extend from the box to show the range of the data.

Female participants had significantly larger tracking errors than male participants (see Fig. 4A). Specifically, the cursor-target distance was 16% greater in women compared to men [hereafter mean ± SD; F = 1.87 ± 0.42 vs. M = 1.62 ± 0.30 cm; t(48) = 2.43; *p* = 0.02]. The effect size (Cohen’s d) was 0.69. Second, we investigated the accuracy of manual tracking at the temporal level by means of crosscorrelation techniques. Estimated time-lag corresponds to the shift needed to maximize cross-correlation coefficients. Notice that hand-cursor cross-correlations were always largely significant (R > 0.55; p < 0.001) with no differences between males and females cross-correlation coefficients (M = 0.9719 vs. F = 0.9718). We found that female hand movements were lagging more behind target motion than was observed for male participants (see Fig. 4B). Indeed, the cursor-target lag was 22 ms longer in women compared to men [F = 71 ± 25 vs. M = 49 ± 21 ms; *t*(48) = 3.46; *p* = 0.001]. The effect size (d) was 0.98. Importantly when realigning hand and target signals so as to compensate for temporal delays (see Fig. 4C), the residual tracking error became similar in men and women [M = 1.40 ± 0.28 vs. F = 1.50 ± 0.34 cm; *t*(48) = 1.11; *p* = 0.27]. We conclude that this difference in the timing of hand motion timing is indeed crucial for the male advantage exhibited during manual tracking.

### Similar hand movement kinematics in male and female

**Figure 5:**
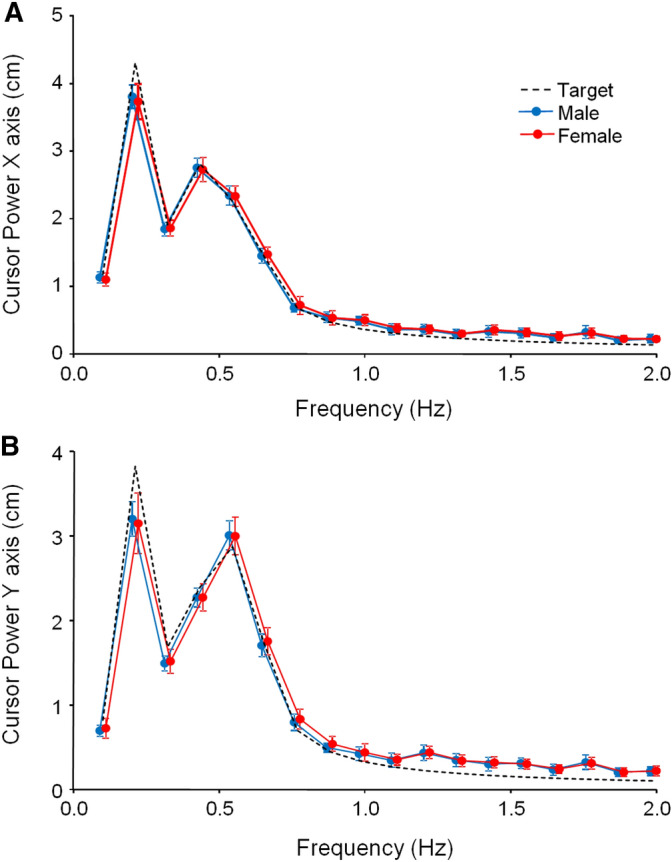
Kinematics of hand movement. (**A**) Spectral analyses of cursor and target motion along the horizontal axis. (**B**) Same as (**A**) for the vertical axis. Males and females use similar hand movements during manual tracking. Error bars, SEM.

As expected spectral analyses showed that the frequency content of cursor motion was largely determined by target motion, however, there was no obvious difference between male and female power spectra (see Fig. 5). For both horizontal and vertical movements, two-way ANOVAs showed no main effect of SEX [*F*(1,48) < 0.015; *p* > 0.60], as well as no SEX by FREQUENCY interaction [*F*(17,816) < 0.01; *p* > 0.49]. The effect of FREQUENCY was significant [*F*(1,44) = 47.55; *p* < 0.001]. We also observed that the characteristics of cursor tangential velocity was very similar in men and women, no matter whether we focused on its mean value [M = 18.5 ± 1.1 vs. F = 18.6 ± 1.4 cm/s; *t*(48) = 0.14; *p* = 0.89; d = 0.04], or its standard deviation [M = 9.67 ± 0.31 vs. F = 9.66 ± 0.30 cm/s; *t*(48) = 0.03; *p* = 0.97; d = 0.01]. Regarding the complexity of hand motion, very similar values of *ApEn* were obtained in men and women, no matter we focused on horizontal [M = 052 ± 0.04 vs. F = 0.52 ± 0.04; *t*(48) = 0.06; *p* = 0.95; d = 0.02], or vertical movements [M = 0.51 ± 0.04 vs. F = 0.50 ± 0.03; *t*(48) = 0.76; *p* = 0.45 ; d = 0.22]. Finally, based on the tangential acceleration of the cursor, the number of hand submovements was similar in men and women [M = 57.9 ± 5.9 vs. F = 59.0 ± 6.5; *t*(48) = 0.61; *p* = 0.54 ; d = 0.17]. Overall, these analyses showed that the greater accuracy exhibited by men during manual tracking is not associated with key differences in hand movement kinematics.

Although instructions given to participants did not require fast initiation of manual tracking at the beginning of each trial, we felt it could be useful to assess whether cursor motion was initiated with a similar latency in men and women. Based on cursor tangential velocity (threshold of 10 cm/s), we found no significant difference between male and female in cursor latency [M = 325 ± 55 vs. F = 329 ± 57 ms; t(48) = 0.26; p = 0.79 ; d = 0.07].

### Similar gaze behavior in male and female

**Figure 6:**
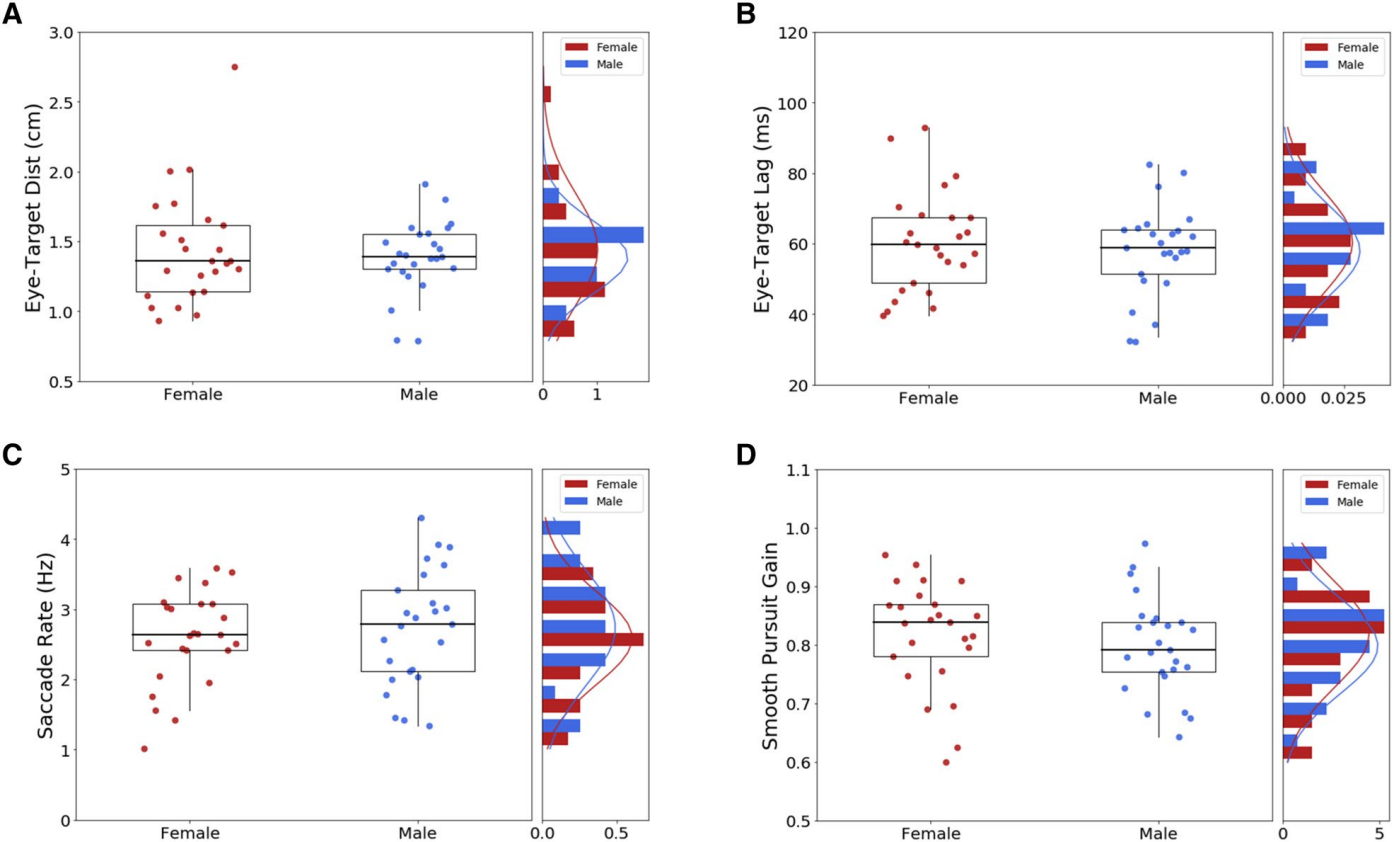
Gaze behavior. (**A**) Eye-target distance. (**B**) Eye-target lag. (**C**) Saccade rate. (**D**) Gain of smooth pursuit. Each box extends from the upper to lower quartile values of the data, with a line at the median. The whiskers extend from the box to show the range of the data. Gaze behavior was nearly identical in males and females.

As the male–female difference in hand tracking could follow from different strategies in sampling visual information, we also performed a detailed analysis of eye movements. However, these analyses revealed no key difference between male and female participants. Although no explicit instruction was given regarding eye movements, as expected gaze was mainly directed to target motion^20,29^. Examining how close the gaze was from the target revealed no obvious sex differences. Indeed no significant difference was found between men and women in terms of eye-target distance [M = 1.39 ± 0.26 vs. F = 1.44 ± 0.40 cm; *t*(48) = 0.60; *p* = 0.55 ; d = 0.17; see Fig. 6A], nor in eye-target lag [M = 58 ± 13 vs. F = 60 ± 14 ms; *t*(48) = 0.63; *p* = 0.53 ; d = 0.17; see Fig. 6B]. Moreover, further examination revealed similar saccade rate [M = 2.73 ± 0.83 vs. F = 2.59 ± 0.67; *t*(48) = 0.68; *p* = 0.50 ; d = 0.19; see Fig. 6C], and similar smooth pursuit gains [M = 0.80 ± 0.08 vs. F = 0.82 ± 0.09; t(48) = 0.80; *p* = 0.43 ; d = 0.23; see Fig. 6D]. Thus, we conclude that the male advantage for manual tracking does not stem from more accurate monitoring of the target with eyes, neither from key changes in saccadic or smooth pursuit activity.

### Eye-hand coordination holds separately in male and female

**Figure 7:**
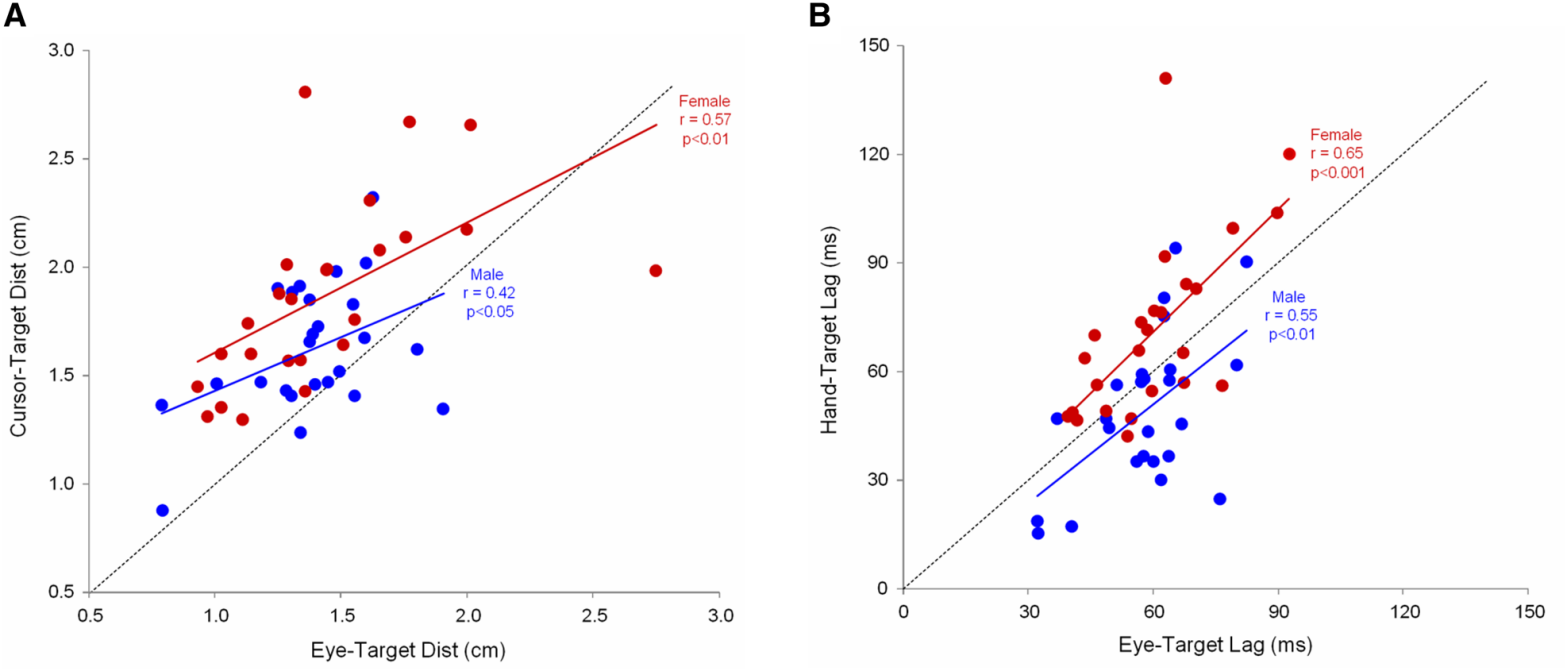
Eye-Hand coordination in terms with respect to target distance (**A**) and target lag (**B**). Distances presented in (**A**) are computed using raw signals (i.e. no lag compensation). The relationship between the accuracy of eye and hand tracking holds separately for males and females.

On the one hand, it is often advocated that there is an intricate relationship between eye and hand movements^29,47,48^. On the other hand, we show here that males and females exhibit a different level of manual skill while using similar gaze behavior. To reconcile those seemingly conflicting observations, we took advantage of interindividual differences and explored the relationship between the accuracy of eye and hand tracking within each group. Figure 7 shows that indeed eye and hand performance are correlated, such that if a participant exhibits accurate eye tracking, he or she is likely to exhibit also accurate manual tracking. However, the presence of an offset along the vertical axis (manual tracking), for both spatial (panel A) and temporal (panel B) errors, shows that this relationship holds separately for males and females. We conclude that, despite the lack of key changes in gaze behavior, the male advantage in manual tracking does not preclude the well-established eye-hand coordination.

### Robustness of a male advantage in manual tracking

**Figure 8:**
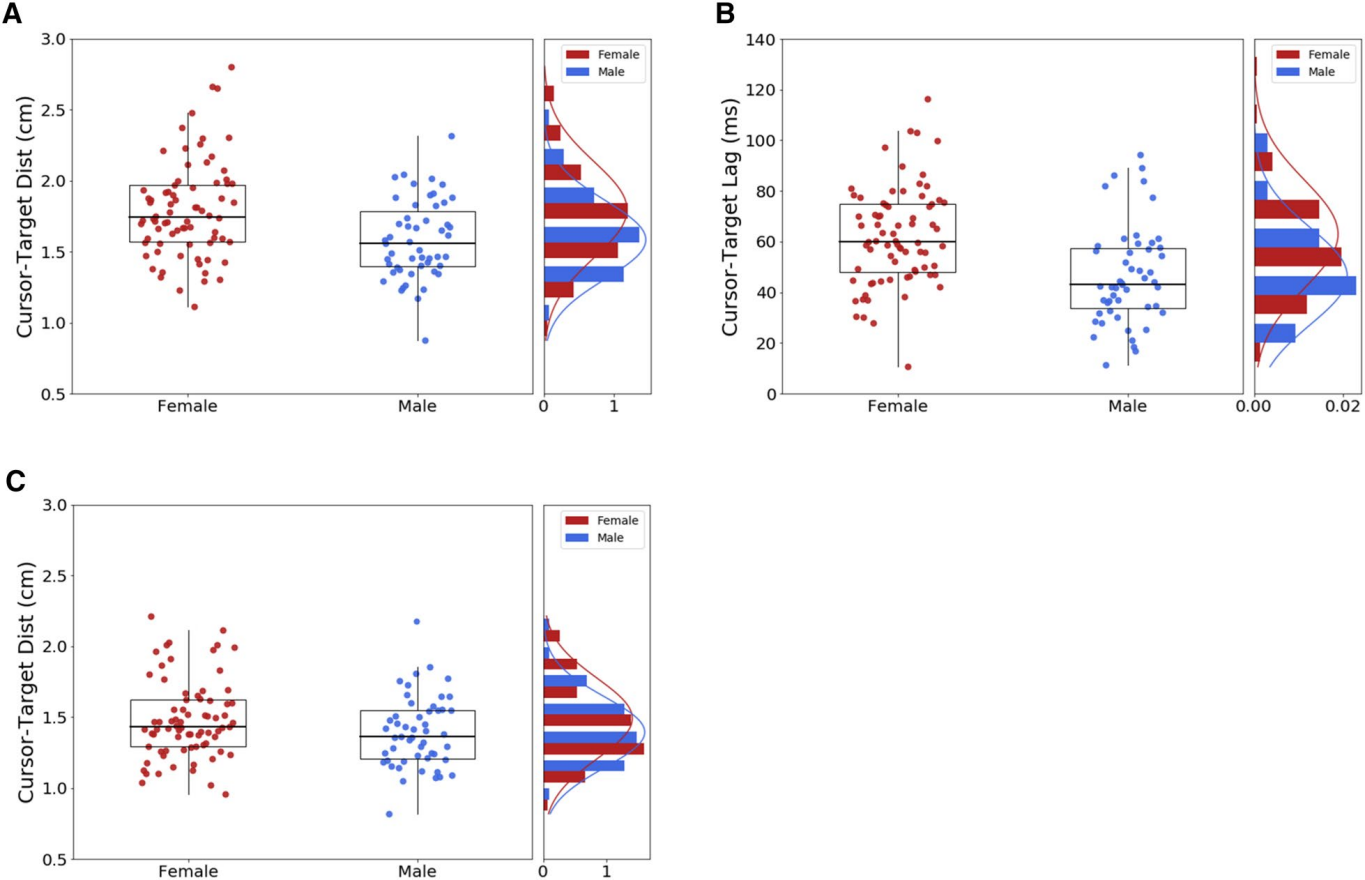
Overall manual tracking performance when pooling the first and second cohort of participants. (**A**) Cursor-target distance. (**B**) Cursor-target lag. (**C**) Residual cursor-target distance after compensation for cursor-target lag. Each box extends from the upper to lower quartile values of the data, with a line at the median. The whiskers extend from the box to show the range of the data.

Given that the male advantage does not stem from a more refined gaze strategy, we took the opportunity to retest this sex difference using our second cohort of 77 naïve participants that performed the very same task except eye movements were not recorded. This second cohort confirmed a male advantage since the cursor-target distance was found to be 13% greater in women than men [F = 1.76 ± 0.29 vs. M = 1.55 ± 0.25 cm; *t*(75) = 3.11; p < 0.001; d = 0.77], along with a larger cursor-target lag in women than men [F = 59 ± 18 vs. M = 42 ± 17 ms; *t*(75) = 3.91; *p* < 0.001; d = 0.95]. Again when compensating for temporal delays, no more sex differences was found, with men and women exhibiting similar cursor-target distance [M = 1.38 ± 0.22 vs. F = 1.48 ± 0.26 ms; *t*(75) = 1.71; *p* = 0.09; d = 0.42]. These analyses conducted on a second pool of participants account for the robustness of our previous observations. Figure 8 presents the overall distribution of manual tracking performance when pooling the two cohorts of participants (127 in total). Again the cursor-target distance was found greater in women than men [F = 1.80 ± 0.34 vs. M = 1.58 ± 0.28 cm; *t*(125) = 3.70; *p* < 0.001; d = 0.68], along with a larger cursor-target lag in women than men [F = 63 ± 21 vs. M = 45 ± 19 ms; *t*(125) = 4.73; *p* < 0.001; d = 0.87], and a lack of sex differences in cursor-target distance after lag compensation [F = 1.48 ± 0.28 ms vs. M = 1.38 ± 0.25 vs.; *t*(125) = 1.94; *p* = 0.06; d = 0.35].

Our experimental design ran over 10 consecutive trials. The difference observed between males and females could be due to a different dynamics of performance over the course of these trials. To investigate such temporal dynamics in more details, we performed a two-way ANOVA (TRIALS × SEX) for each dataset. For both datasets we found a main effect of TRIAL (*p* < 0.001) and SEX (*p* < 0.01). But more important, the SEX by TRIAL interaction never reached significance (*p* > 0.24), thereby demonstrating that the male advantage, as evidenced by a smaller cursor-target lag, is robust over the course of our experimental paradigm.

Finally to further test the robustness of a sex difference, we performed one more analysis. As explained above we carefully matched age distribution between male and female distribution by removing a few (12) participants from our complete database. To discard that such age normalization has not introduced any bias, we repeated our analysis on hand tracking over the full, aggregated database (i.e. 139 participants). Indeed, we obtained identical results with 127 or 139 participants: there was a male advantage in hand tracking, as evidenced by a smaller cursor-target distance [M = 1.62 vs. F = 1.80 cm; *t*(137) = 2.80; *p* < 0.01], and a smaller cursor-target lag [M = 48 vs. F = 63 ms; *t*(137) = 4.13; p < 0.001].

## Discussion

Here we provide strong evidence for a large and robust difference in visuomotor tracking performance between males and females, as evidenced by a smaller cursor-target distance in males, resulting from a smaller cursor-target lag. In both cases, the male advantage was in the medium to large effect size range (from 0.69 up to 1.25), which are as strong as the largest sex differences reported in other domains^3,31,49^. Our results fit well with earlier studies investigating the effect of sex on manual tracking, even though experimental setups could vary^16–19^.

Importantly, our detailed analyses of hand kinematics suggest that the male advantage in our visuomotor task cannot be explained by the fact that males would produce more sophisticated hand movements. Indeed, no key difference was found between men and women from the spectral analysis of hand motion trajectories, nor when we explored the smoothness, intermittency or complexity of hand movement kinematics. Obviously, we are aware of a male advantage for hand muscle strength as well as sex differences in muscle endurance^50–52^, but it is doubtful how these motor factors may have contributed to better tracking performance in men. First, the lever of the joystick had very low inertia and was not even spring-loaded. Second, in contrast to motor tasks that require fast and full arm movements (e.g. throwing, pegboard, box and block), our task required rather slow (< 5 cm/s) and minimal (< 5 cm) excursion of the hand. Overall, the greater muscle strength and endurance of men are unlikely to account for their greater accuracy in manual tracking.

Considering that gaze provides salient information during visually guided actions, we also examined eye movements. However our results were clear, men and women exhibit similar gaze strategies during manual tracking. Not only we failed to demonstrate reliable sex differences in the accuracy of eye tracking, but we also did not find any sex differences regarding oculomotor properties, with both saccades and smooth pursuit characteristics being similar between the two groups. Overall we conclude that men and women had presumably access to similar retinal and extra-retinal signals when planning their upcoming hand movements.

Having shown that the male advantage for manual tracking is not associated with key differences in hand movement kinematics or gaze behavior, we examined the effectiveness of hand movements in the temporal domain. First, we found that hand movements were lagging more on the target in women than men, an effect that was on the order of 20 ms. Second, we showed that this 20 ms delay accounted for most of the sex differences in manual tracking accuracy. Third, sex differences in cursor-target lag did not merely originate from sex differences in initiating the task. We reason that, in our tracking task, participants were constantly guessing the next target state so as to accommodate their ongoing hand and eye movements accordingly. Given that males and females exhibited similar eye tracking performance (same eye-target distance and lag), their ability to predict the target dynamics would be presumably similar. Although this may seem like a reasonable conclusion, this brings a new question: if men and women make similar guesses about future target states, why does the hand lag more on the target in women than men? To reconcile these conflicting observations we propose that the decisional process linking visual information about cursor and target with forthcoming hand actions is similarly accurate in men and women but simply takes a bit longer in women. This proposition echoes with earlier studies showing that women exhibit slower reaction times to visual stimuli than men^36–40^, which on average were 20 ms longer. Moreover, it is worth reporting that anatomical and functional sex differences have been reported in the cerebellum^53,54^, a key structure for eye-hand coordination^8,55–57^. Specifically, brain imaging has revealed that the vermis and the cerebellar hemispheres were larger in men, even after taking into account differences in body size^53^. Furthermore, it has been reported greater inter-hemispheric connectivity between the lobes of the cerebellum in men^54^. Although a causal relationship between the size (or connectivity) of the cerebellum and the efficiency of eye-hand coordination is not yet established, the possibility that the male advantage found in the current study could follow from sex differences in the cerebellum seems worth exploring.

Although our results indicate a male advantage for manual tracking, sex differences must be interpreted cautiously, as many other (hidden) factors may encourage differences between men and women^58^. Indeed, we cannot exclude the possible contribution of social and environmental factors in the emergence of a male advantage. For instance, it has been suggested some of these sex differences could stem from different levels of expertise/training in video-games. Indeed, it has been previously reported that regular practice in video-games provides some advantages for visuomotor tracking^59^, eye-hand coordination^60^, as well as a general speeding of reaction times without decreases in accuracy^61^. Considering that women are less inclined to play video-games than men^62^, this may have encouraged the advantage exhibited by men seen in our task (see also^59^). Although the current study did not assess participants’ level in video games, there are still some concerns with this line of reasoning. First, some studies reporting a male advantage in manual tracking^17^ and reaction time^36,40^ were performed before the birth of video-games. Second, a recent study performed with men and women having limited experience in video games showed a male advantage in a complex spatially-demanding video game^63^. Moreover, although sex differences diminished over the course of training, this study showed that the male advantage persisted even after 30 h of practice. In the current study, we found no evidence that the male advantage tended to fade away across the course of ten trials. Thus, the male advantage observed in the temporal accuracy of manual tracking appears to be both genuine across trials and robust across a large set of participants. Still, future studies confirming whether this observation holds over a larger set of trials would certainly be helpful.

## Conclusions

In summary, the current study provides clear evidence of a difference in visuomotor processing between males and females. Our results show that this male advantage does not reside in a more refined gaze strategy, or more sophisticated hand movements, but rather in a faster decisional process linking visual information of the target with forthcoming hand actions. More generally there is a growing interest in sex differences in funding agencies, the neuroscience community, as well as in medicine for drug treatment and rehabilitation strategies^6^. The current study reinforces the view that incorporating sex as a biological variable is relevant in behavioral neuroscience^4,5^.

## Data Availability

Data are available at the following site: https://zenodo.org/record/3903369
